# Long non-coding RNAs: Modulators of phenotypic transformation in vascular smooth muscle cells

**DOI:** 10.3389/fcvm.2022.959955

**Published:** 2022-08-26

**Authors:** Bing-Han Lu, Hui-Bing Liu, Shu-Xun Guo, Jie Zhang, Dong-Xu Li, Zhi-Gang Chen, Fei Lin, Guo-An Zhao

**Affiliations:** ^1^Department of Cardiology, Life Science Center, The First Affiliated Hospital of Xinxiang Medical University, Weihui, China; ^2^Key Laboratory of Cardiovascular Injury and Repair Medicine of Henan, Weihui, China; ^3^Henan Normal University, Xinxiang, China

**Keywords:** long non-coding RNAs, vascular smooth muscle cells, phenotypic transformation, vascular disease, atherosclerosis

## Abstract

Long non-coding RNA (lncRNAs) are longer than 200 nucleotides and cannot encode proteins but can regulate the expression of genes through epigenetic, transcriptional, and post-transcriptional modifications. The pathophysiology of smooth muscle cells can lead to many vascular diseases, and studies have shown that lncRNAs can regulate the phenotypic conversion of smooth muscle cells so that smooth muscle cells proliferate, migrate, and undergo apoptosis, thereby affecting the development and prognosis of vascular diseases. This review discusses the molecular mechanisms of lncRNA as a signal, bait, stent, guide, and other functions to regulate the phenotypic conversion of vascular smooth muscle cells, and summarizes the role of lncRNAs in regulating vascular smooth muscle cells in atherosclerosis, hypertension, aortic dissection, vascular restenosis, and aneurysms, providing new ideas for the diagnosis and treatment of vascular diseases.

## Introduction

**Figure 1:**
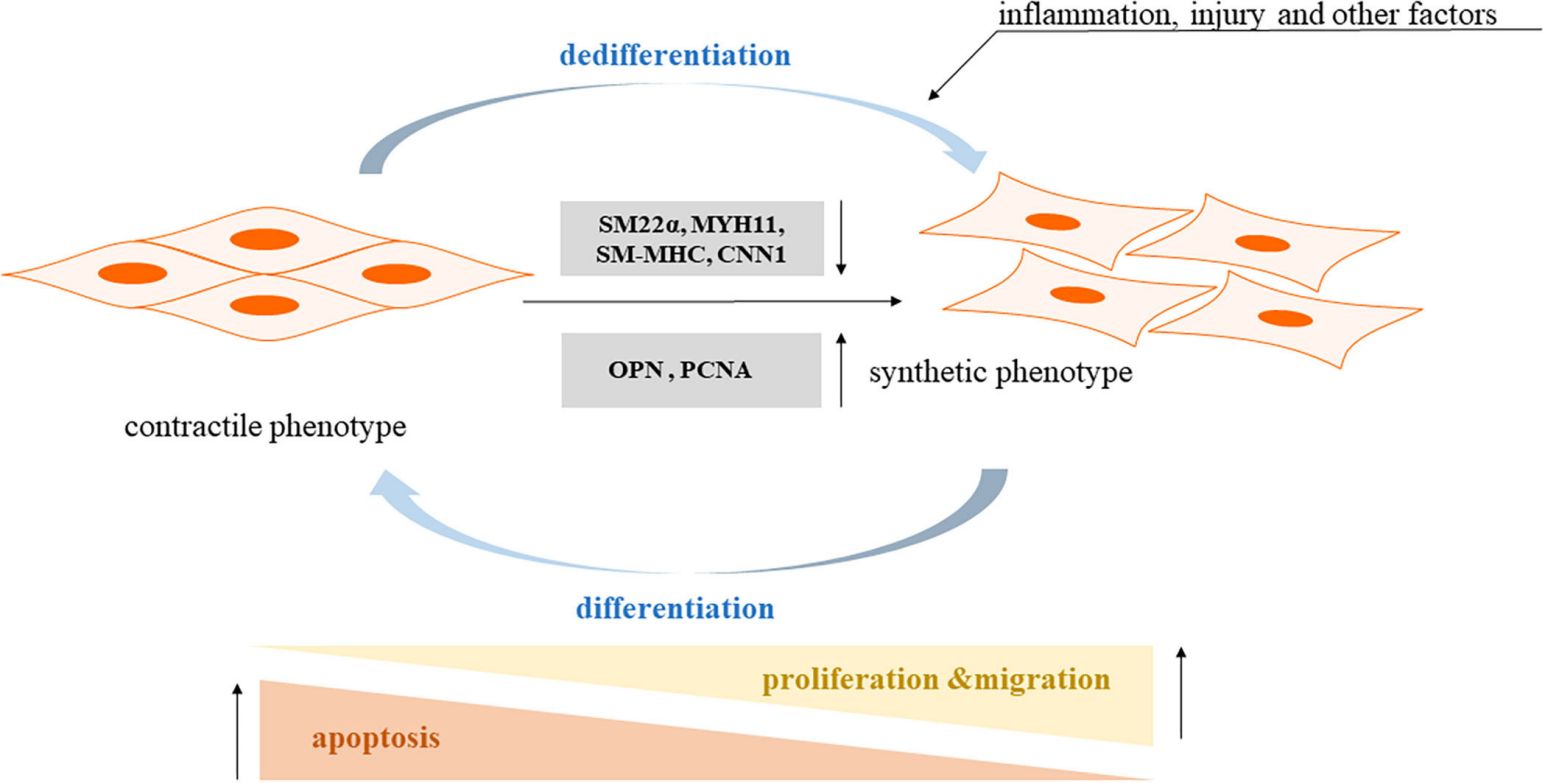
The pathophysiological mechanism of smooth muscle cells phenotypic transformation. Contractile phenotype markers: SM22a: smooth muscle 22a; SM-MHC: smooth muscle myosin heavy chain; MYH11: myosin heavy chain 11; CNN1: calponin 1. Synthetic phenotype markers: OPN: osteopontin; PCNA: proliferating cell nuclear antigen.

Phenotype transformation of vascular smooth muscle cells (VSMCs) is an important cause of vascular dysfunction, capable of inducing vascular diseases, such as atherosclerosis (AS), hypertension, vascular stenosis, and diabetic vascular complications (1–6), and mature smooth muscle cells are widely distributed in the walls of blood vessels and internal organs, and normal VSMCs have no significant function in proliferating, migrating, and secreting the extracellular matrix, called constrictive VSMCs, which maintain vascular elasticity and ensure vasoconstriction (7). VSMCs exhibit significant proliferation and migration under immature or pathological conditions, such as inflammation, hypertension, and diabetes, and they synthesize large amounts of extracellular matrix, which are called synthetic VSMCs (8). After the phenotypic transformation occurs in smooth muscle cells, they change from “contractile type” to “synthetic type,” causing changes in vascular function and playing an important role in the development of vascular remodeling, and increasing evidence is emerging that the phenotype of VSMCs can develop fibroblastic, osteoblastic, and even macrophage-like cell characteristics (9, 10). Thus, understanding the pathophysiological changes in muscle cells is essential for diagnosing and treating vascular diseases. For a better understanding, we have made a graph, as shown in Figure 1.

**Figure 2:**
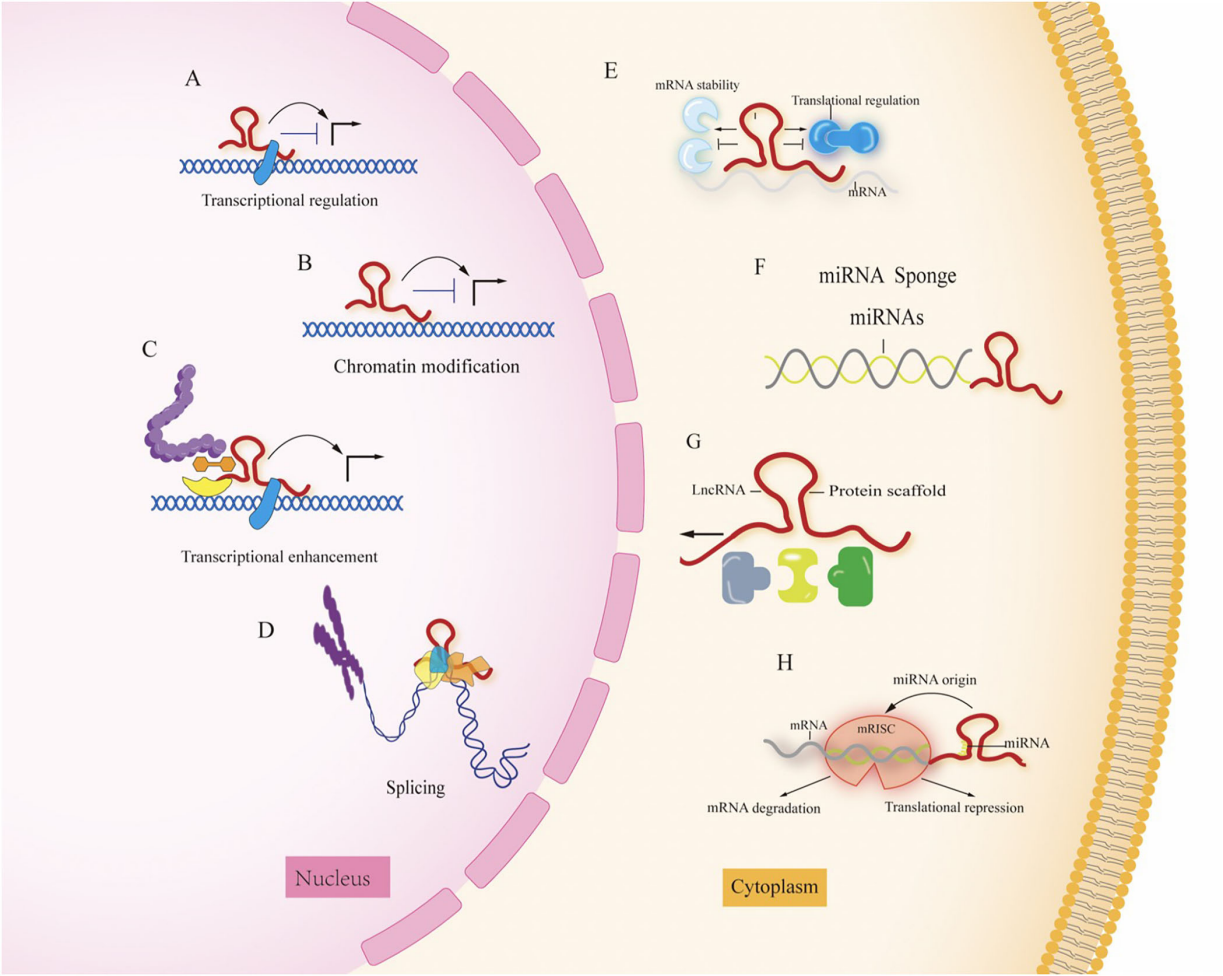
The different roles of LncRNA in the nucleus and cytoplasm. **(A)** Transcriptional regulation; **(B)** chromation modification; **(C)** transcriptional enhancement; **(D)** splicing; **(E)** regulation of mRNA stability; **(F)** miRNA sponge; **(G)** protein scaffold; **(H)** LncRNAs can function as miRNA host transcripts (miRNA origin). Mature single-stranded miRNA is derived from lncRNA transcripts and loaded into RNA induced silencing complex (RISC) by repressing translation or inducing mRNA degradation.

Long non-coding RNAs (lncRNAs) are longer than 200 nucleotides and cannot code for proteins (11); according to the genome and the location relationship between adjacent genes, lncRNAs can be divided into sense, antisense, bidirectional, intronic, and intergenic lncRNA (12), and lncRNA expression has the spatial specificity of tissue expression. They play important roles in disease development, such as regulating transcription, epigenetic modifications, protein, and RNA stability, and translation and post-translational modifications, by interacting with DNA, RNA, and proteins, which are closely related to their intracellular localization, lncRNAs localized in the nucleus play several roles: (1) regulate chromatin remodeling, induce histone modifications to regulate downstream gene expression; (2) act as enhancer RNAs to regulate transcription; and (3) interfere with pre-mRNA processing to regulate mRNA splicing. LncRNAs localized in the cytoplasm can play several roles: (1) act as decoys that can regulate specific transcription factors and inhibit their function (13, 14); (2) act as sponges to adsorb miRNAs, regulate their stability and reduce their bioavailability (15); (3) act as molecular scaffolds that can bring two or more proteins into complex transcriptional or post-transcriptional complexes regulating gene expression; and (4) binding to specific proteins to affect protein translation and post-translational modifications, or as a precursor molecule for small molecule RNAs (16). The specific functions are shown in Figure 2 (17).

LncRNAs regulate smooth muscle cell phenotypic transition (18) and play a key role in related diseases, but the molecular mechanisms are not fully understood. In this review, we briefly outline the effect of the regulation of lncRNAs on differentiation and phenotypic transition in VSMCs during pathological remodeling. We also focus on how lncRNAs play a regulatory role in various conditions and their contribution to vascular diseases.

## Role of LncRNA in smooth muscle cell phenotypic transformation

This review focuses on the mechanisms by which LncRNAs are known to play a regulatory role in various conditions and their contribution to vascular diseases. Table 1 shows the lncRNAs implicated in the regulation of VSMC phenotype and their validated targets.

**Table 1:** Long non-coding RNA with functional relevance in different vascular diseases.

**LncRNA**	**Regulation**	**Related target**	**The function in the VSMCs**	**Disease**	**References**	**Genbank accession numbers**
SMILR	Promote	HAS2	Proliferation	Atherosclerosis	Zhang et al. (19), Ballantyne et al. (20)	105375734
P21	Inhibit	p53, p300, miR-17-5p	Proliferation, apoptosis	Atherosclerosis	Wu et al. (21), Wang et al. (22)	102800311
ANRIL	Inhibit	AMPK, WDR5, HDAC3, miR-126-5p	Proliferation, apoptosis	Atherosclerosis, PAH	Zhang et al. (23), Li et al. (24), Wang et al. (25)	100048912
ZNF800	Inhibit	PTEN, AKT/mTOR	Proliferation, migration	Atherosclerosis	Lu et al. (26)	168850
MIAT	Promote	EGR1-ELK1-ERK, miR-641	Proliferation, migration, invade	Atherosclerosis	Fasolo et al. (27), Ma et al. (28)	440823
430945	Promote	RhoA	Proliferation, migration	Atherosclerosis	Cui et al. (29)	23569
BANCR	Promote	miR-34c	Proliferation, apoptosis	Atherosclerosis	Jiang et al. (30)	100885775
RP11-531A24.3	Inhibit	ANXA2	Proliferation, migration	Atherosclerosis	Wu et al. (31)	26121
CARMN	Inhibit	MYOCD	Proliferation, migration	Atherosclerosis	Onuh et al. (32), Miano, (33), Dong et al. (34).	728264
PEBP1P2	Inhibit	CDK9	Proliferation, migration	Atherosclerosis	He et al. (35)	647307
LIPCAR	Promote	P21, CDK2	Proliferation, migration	Atherosclerosis	Hung et al. (36), Wang et al. (37)	103504742
H19	Promote	miR-675, miR-599, miR-148b, miR-193b-3p	Proliferation, migration, apoptosis	Atherosclerosis, AD	Cai et al. (38), Sun et al. (39), Lu et al. (40), Zhang et al. (41), Lv et al. (42), Ren et al. (43)	283120
AC105942.1	Inhibit	hnRNPA2/ B1	Proliferation	Atherosclerosis	Zhang et al. (44)	2157
TUG1	Promote	miR-21	Proliferation	Atherosclerosis	Li et al. (45)	55000
FOXC2-AS1	Promote	miR-1253	Proliferation, apoptosis	Atherosclerosis	Wang et al. (46)	103752587
HCG11	Promote	miR-144	Proliferation, apoptosis	Atherosclerosis	Liu et al. (47)	493812
CTB P1-AS2	Inhibit	miR-195-5p	Migration	Atherosclerosis	Wang et al. (48)	92070
XIST	Promote	miR-539-5p, miR-17	Proliferation, migration, invade	Atherosclerosis, TAAD	Wang et al. (48), Zhang et al. (49)	7503
CASC2	Inhibit	miR-532-3p	Proliferation, apoptosis	Atherosclerosis,PAH	Wang et al. (50), Gong et al. (51)	255082
MEG3	Promote	miR-361-5p	Proliferation, apoptosis	Atherosclerosis	Wang et al. (52)	55384
MEG8	Inhibit	miR-181a-5p, miR-195-5p	Proliferation, migration, apoptosis	Atherosclerosis	Zhang et al. (53), Xu et al. (54)	79104
MALAT1	Inhibit	miR-124-3p	Proliferation, apoptosis	Atherosclerosis	Cheng et al. (55)	378938
SNHG7-003	Inhibit	miR-1306-5p	Proliferation, migration, invade	Atherosclerosis	Zheng et al. (56)	84973
C2dat1	Promote	miR-34a	Proliferation, migration	Atherosclerosis	Wang et al. (57)	107980436
SNHG12	Inhibit	miR-7665p, miR-199a-5p	Proliferation, migration	Atherosclerosis	Liu et al. (58), Sun et al. (59)	85028
LEF1-AS1	Promote	miR-544	Proliferation, migration, invade	Atherosclerosis	Zhang et al. (60)	641518
01123	Promote	miR-1277-5p	Proliferation, migration	Atherosclerosis	Weng et al. (61)	440894
00341	Promote	miR-214	Proliferation, migration	Atherosclerosis	Liu et al. (62)	79686
ES3	Promote	miR-95-5p, miR-6776-5p, miR-3620-5p and miR-4747-5p	Osteoblast-like cells	Diabetes	Zhong et al. (63)	100507428
EPS	Inhibit	Wnt/β-catenin	Migration,osteoblast-like cells	Diabetes	Li et al. (64)	102635290
UCA1	Promote	miR582-5p, hnRNP I	Proliferation, apoptosis, invade	Diabetes, PAH	Yang et al. (65), Zhu et al. (66)	652995
HCG18	Inhibit	fused in sarcoma (FUS)	Proliferation, apoptosis	Hypertension	Lu et al. (67)	414777
GAS5	Inhibit	miR-21, p53, NOXA	Proliferation, migration	Hypertension, restenosis	Liu et al. (68), Tang et al. (69).	60674
MRAK048635_P1	Inhibit	Rb,E2F	Proliferation, migration, apoptosis	Hypertension	Fang et al. (70)	25102670
AK098656	Promote	MYH11/ FN1	Proliferation	Hypertension	Jin et al. (71).	831169
CDKN2B-AS1	Promote	miR-143-3p	Proliferation, migration	Restenosis	Ma et al. (72).	100048912
CRNDE	Promote	smad3	Proliferation, migration	Restenosis, AAA	Zhou et al. (73), Li et al. (74).	643911
NEAT1	Promote	WDR5,miR-34a-5p, KLF4	Proliferation, migration	Restenosis, PAH	Ahmed et al. (75), Dou et al. (76).	283131
Hoxaas3	Inhibit	H3K9,Hoxa3	proliferation	PAH	Zhang et al. (77).	72628
TCONS_00034812	Inhibit	STOX1	Proliferation	PAH	Liu et al. (78).	100506542
Rps4l	Inhibit	ILF3	Proliferation, migration	PAH	Liu et al. (79)	66184
AC068039.4	Promote	miR-26a-5p	Proliferation, migration	PAH	Qin et al. (80)	10982
MYOSLID	Inhibit	Smad2,MKL1	Proliferation, migration	PAH	Zhao et al. (81)	105373853
01278	Inhibit	miR- 500b-5p	Proliferation, migration	AD	Wang et al. (82)	92249
PVT1	Promote	miR-27b-3p,miR-3127-5p	Proliferation, migration	AD, AAA	Li et al. (83), Huang et al. (84)	5820
LUCAT1	Inhibit	miR-199a-5p	Proliferation, apoptosis	AAA	Xia et al. (85)	100505994
SNHG5	Promote	miR-205-5p	Proliferation, migration, apoptosis	AAA	Nie et al. (86)	387066
00473	Inhibit	miR-212-5p	Proliferation, apoptosis	AAA	Tian et al. (87)	90632

LncRNAs and their regulation (promote or inhibit) under vascular disease-relevant conditions. Further depicted in the table are the downstream targets of lncRNAs, their main function in VSMC dynamics, the respective references, and the GenBank accession numbers. Abbreviations of target genes are explained in the text.

### LncRNA and atherosclerosis

Dysfunction of smooth muscle cells can trigger plaque formation, which is an important link in the pathogenesis of atherosclerosis, namely, the phenotypic transformation of proliferation, migration, and apoptosis. Platelet-derived growth factor-BB (PDGF-BB), oxidized low-density lipoprotein (ox-LDL), interleukin-6 (IL-6), and tumor necrosis factor-α (TNF-α) can change the phenotypic transformation of VSMCs from a contractile to a synthetic phenotype, promote smooth muscle cell proliferation and migration, and inhibit apoptosis. Matrix metalloprotein 2 (MMP-2) and matrix metalloprotein 9 (MMP-9) are important VSMC migration regulators. B cell lymphoma-2 (Bcl-2) and BCL2-Associated X (Bax) are apoptosis-related proteins. LncRNAs play various roles in regulating smooth muscle cell phenotypic transformation, as well as cell proliferation and migration. Detection of smooth muscle cell phenotypic transformation-related markers, cell proliferation, apoptosis-related proteins, and other clear regulatory roles implies that the effective control of smooth muscle cell phenotypic transformation may be an important therapeutic measure to prevent and treat AS.

The lncRNA SMILR can act as an enhancer or molecular scaffold to promote the proliferation of VSMCs by interacting with the promoter region of hyaluronidase 2 (HAS2), an important component of the extracellular matrix deposited in AS lesions, which promotes vessel wall thickening and reflects the degree of AS disease progression (19). Animal studies show that VSMC-specific HAS2 overexpression in transgenic mice increases susceptibility to AS and promoted vessel wall thickening. The investigators found increased expression of lncRNA SMILR in unstable AS plaques, which was detectable in the plasma of patients. These results confirmed that lncRNA SMILR is a driver of VSMC proliferation (20). However, it has been shown that lincRNA-p21 can also act as an enhancer partially bound to mouse double minutes2 (MDM2), enhancing the transcriptional activity of p53 and enabling p53 to interact with protein 300 (p300) and bind to the promoter/enhancer of its target gene, thereby inhibiting cell proliferation and inducing apoptosis in VSMCs (21). Another study showed that under ox-LDL stimulation, lncRNA antisense non-coding RNA in the INK4 locus (ANRIL) can act as a molecular scaffold to promote the binding of WD-40 repeat-containing protein 5(WDR5) and histone deacetylase 3 (HDAC3), thus, forming a WDR5/HDAC3 complex that regulates the expression of the target gene NADPH oxidase 1 (NOX1) through histone modifications, upregulates reactive oxygen species (ROS) levels, promotes phenotypic transition in HASMCs, and is a potential scaffolding protein (23).

LncRNAs can exert a role by directly binding to proteins and participating in protein phosphorylation and the activation of signaling pathways. lncRNAs are required to localize specific protein complexes, which can interact with DNA or mRNA and inhibit their expression or translation through methylation. In AS plaques, lncRNA ZNF800 expression is upregulated by directly binding to phosphatase and tensin homolog deleted on chromosome 10 (PTEN), thereby blocking the AKT (also known as protein kinase B PKB)/mammalian target of rapamycin (mTOR) pathway to inhibit PDGF-BB-mediated proliferation and migration of VSMCs. MMP1 promotes cell migration by degrading ECM components, and vascular endothelial growth factor-α (VEGF-α) can also lead to cell proliferation and migration. LncRNA ZNF800 regulates the hypoxia-inducible factor-1α (HIF-1α)-mediated VEGF-α or MMP1 pathway through the PTEN-activated AKT/mTOR signaling pathway to inhibit VSMC proliferation and migration (26). Similarly, the inhibition of lncRNA myocardial infarction-associated transcript (MIAT) limits the phosphorylation of extracellular signal-regulated kinase (p-ERK), increases the phosphorylation of ETS transcription factor (p-ELK1) accumulation in the nucleus, and subsequently decreases early growth response 1 (EGR1) expression, thereby regulating the proliferation of smooth muscle cells (SMCs) through the EGR1-ELK1-ERK pathway. The lncRNA MIAT also binds to the promoter region of Krüppel-like factor 4 (KLF4) and enhances its transcription, participating in the phenotypic transformation of SMCs to pro-inflammatory macrophage-like cells. *In vivo* studies have shown that SMCs in mouse and minipig models of AS display changes similar to those of HASMCs, thus confirming that lncRNA MIAT plays a regulatory role in advanced AS lesion formation by inducing the differentiation and dedifferentiation of SMCs (27). Another study showed that lncRNA 430945 is highly expressed in human AS tissues, which in turn promotes the angiotensin II (AngII)-induced proliferation of VSMCs. The upregulation of lncRNA 430945 expression activates signaling pathways associated with receptor tyrosine kinase-like orphan receptor 2 (ROR2) and Ras homolog gene family member A (RhoA), promoting AngII-induced proliferation and migration of VSMCs (29). In contrast, metformin exerts an anti-AS effect by activating AMP-activated protein kinase (AMPK), increasing the expression of lncRNA ANRIL, enhancing the affinity of lncRNA ANRIL to the AMPKγ subunit, increasing the catalytic activity of AMPK, and increasing its phosphorylation level, thereby inhibiting the phenotypic transition of VSMCs (88). The downregulation of miR-34c expression may be owing to the demethylation associated with lncRNA BRAF-activated non-coding RNA (BANCR). High mobility group protein B1 (HMGB1) is a pro-inflammatory mediator that upregulates the expression of cytokines, chemokines, and adhesion molecules, thereby enhancing macrophage infiltration, leading to AS. miR-34c overexpression inhibits the expression of HMGB1, TNF-α, and Bcl-2. LncRNA BANCR overexpression induces HASMC proliferation by downregulating miR-34c methylation and reversing the effect of miR-34c on HMGB1, TNF-α, and Bcl-2 expression, thereby promoting HASMCs proliferation and inhibiting apoptosis (30). The expression of LncRNA RP11-531A24.3 is reduced in advanced AS lesions; in cells overexpressing it, lncRNA RP11-531A24.3 inhibits the migration and proliferation of HA-VSMCs by binding directly to the RNA-binding protein annexin 2 (ANXA2) in the cytoplasm to reduce its expression at the mRNA and protein levels (31).

The cardiac mesoderm enhancer-associated non-coding RNA (CARMN) regulates specific transcription factors, and serum response factor (SRF), a transcription factor that binds to CArG elements, plays an important role in regulating the VSMC phenotype by interacting with multiple cofactors (32). Myocardin (MYOCD) is a specific transcriptional co-activator involved in the differentiation of cardiomyocytes and VSMCs. MYOCD enhances the binding of SRF to the CArG box and transcriptionally activates a variety of downstream VSMC contractile genes, representing the contractile and differentiated VSMC phenotype (33). The lncRNA CARMN enhances trans-MYOCD function by directly binding to MYOCD to maintain the contractile phenotype of VSMCs in healthy arteries. In contrast, in diseased arteries, lncRNA CARMN expression is downregulated, thereby attenuating the trans-activating activity of the MYOCD/SRF complex on SMC-specific gene expression and triggering the dedifferentiation of VSMCs, leading to increased neointimal formation (34). Another transcriptional regulator, cyclin-dependent kinase 9 (CDK9), was shown to be a direct target of lncRNA PEBP1P2, and overexpression of lncRNA PEBP1P2 significantly inhibited proliferation, migration, and dedifferentiation during PDGF-BB-induced phenotypic transformation of VSMCs by directly binding to CDK9 to downregulate its expression. This idea was similarly validated in animal experiments, where lncRNA PEBP1P2 overexpression attenuated neointima formation and VSMC phenotypic transformation induced in a balloon-injured carotid artery model (35). LncRNA LIPCAR accelerates the cell cycle by inhibiting the expression of the anti-proliferative gene P21 and activating the transcriptional regulator CDK2, decreasing the expression of α-SMA, and increasing the expression of MMP-2 and MMP-9 to promote VSMC proliferation, migration, and ultimately endothelial hyperplasia and AS plaque formation (36, 37). In another study, lncRNA H19 was expressed in the neoplastic endothelium of a mouse balloon injury model as well as in the VSMCs of human plaques (38), and knockdown of lncRNA H19 enhanced the interaction between Bax and p53 proteins by increasing p53-regulated transcription, leading to the proliferation of VSMCs and a reduction in plaque size, and mediated VSMC apoptosis to delay the development of AS (39). In AS plaques, lncRNA AC105942.1 expression was downregulated and hnRNPA2/B1 expression was upregulated, whereas hnRNPA2/B1 functions in the cell cycle by regulating the transcriptional levels of cell cycle protein kinase (CDK4) and p27. When lncRNA AC105942.1 expression was upregulated, the proliferation of AngII-treated VSMCs was reduced, CDK4 expression was decreased, and p27 was upregulated, whereas heterogeneous nuclear ribonucleoprotein A2/B1 (hnRNPA2/B1) expression was reduced. hnRNPA2/B1 knockdown also significantly reduced CDK4 expression and upregulated p27 levels, and the results suggest that lncRNA AC105942.1 acts by downregulating hnRNPA2/B1 expression to regulate the transcriptional levels of CDK4 and p27, thereby inhibiting the proliferative effects of AngII on VSMCs (44).

However, in ox-LDL-induced HA-VSMCs, knockdown of lncRNA H19 also acts as a sponge to adsorb miR-599 to reduce pappalysin 1 (PAPPA) to inhibit the increase in cyclin D1 and N-cadherin in HA-VSMCs and decrease E-cadherin to promote proliferation, migration, and invasion of HA-VSMCs (40). H19 also acts as a competitive endogenous RNA (ceRNA) for miR-148b to enhance the expression of wnt family member 1 (WNT1). Moreover, miR-148 inhibitors exert their pro-proliferative and anti-apoptotic effects by activating ox-LDL-stimulated Wnt/β-catenin signaling in HA-VSMCs (41). LncRNA taurine upregulated gene 1 (TUG1) expression was also upregulated in VSMCs induced by hypoxia or TNF-α in patients with AS. In established injury models, lncRNA TUG1 promotes VSMC proliferation and AS by targeting miRNA-21 to downregulate PTEN expression, decrease PTEN activity, and increase cyclin D1 expression (45). Another lncRNA, ANRIL, also called cyclin-dependent kinase inhibitor 2B antisense RNA 1 (CDKN2B-AS1), acts as a ceRNA to competitively bind miR-126-5p to upregulate protein tyrosine phosphatase non-receptor type 7 (PTPN7) expression and inhibit the phosphatidylinositide 3-kinases (PI3K)-AKT pathway, thereby hindering ox-LDL-induced proliferation and accelerating apoptosis (24). LncRNA forkhead box protein C2-AS1 (FOXC2-AS1) expression was significantly upregulated in VSMCs induced by ox-LDL and IL-6. LncRNA FOXC2-AS1 binds to miR-1253 as a ceRNA, causing miR-1253 to target forkhead box protein F1 (FOXF1), increasing the levels of Bcl-2 and significantly decreasing Bax and caspase-3, thereby regulating cell proliferation and the development of AS (46). Similarly, overexpression of lncRNA HLA complex group 11 (HCG11) can act as a sponge to negatively regulate miR-144 while increasing FOXF1 expression, resulting in increased Bcl-2 and decreased Bax expression, thereby promoting proliferation and inhibiting apoptosis in VSMCs (47). Silencing the lncRNA MIAT acts as a sponge for miR-641, induces stromal interaction molecule 1 (STIM1), attenuates the protein expression of proliferating cell nuclear antigen (PCNA), and Ki-67, and thus inhibits ox-LDL-induced proliferation, migration, and invasion (28). Likewise, overexpression of lncRNA C-terminal binding protein 1-antisense RNA 2 (CTBP1-AS2) acts as a ceRNA for miR-195-5p to promote autophagy-related 14 (ATG14) expression and decrease PCNA and Ki-67 expression levels, thereby inhibiting HAVSMC proliferation (48). Downregulation of lncRNA X-inactive-specific transcript (XIST), as a competitive endogenous RNA for miR-539-5p to enhance the expression of secreted phosphoprotein 1, inhibited the upregulation of PCNA and Ki-67 expression, as well as the expression of MMP-2 and MMP-9, thereby suppressing the proliferation, migration, and invasion of VSMCs by ox-LDL stimulation (89). Similarly, overexpression of another lncRNA, cancer susceptibility candidate 2 (CASC2), can act as a sponge to negatively regulate the expression of miR-532-3p, upregulate the expression of non-canonical poly (A) polymerase 5 (PAPD5), and inhibit the expression of PCNA, α-SMA, MMP-2, and MMP-9. LncRNA CASC2 inhibits the proliferation of VSMCs and promotes apoptosis by regulating the miR-532-3p/PAPD5 axis (50). miR-361-5p targets the 3′-UTR of ATP-binding cassette transporter A1 (ABCA1) mRNA and downregulates lncRNA maternally expressed gene 3 (MEG3), possibly by binding miR-361-5p to act as an endogenous “sponge,” thereby abolishing the miRNA-mediated inhibitory activity on the 3′-UTR of ABCA1 and promoting the proliferation and slowing the apoptosis of VSMCs (52). Likewise, overexpression of lncRNA maternally expressed gene 8 (MEG8) indirectly targets peroxisome proliferator-activated receptor α (PPARα) by adsorbing miR-181a-5p at the 3′-UTR and positively regulating its expression, thereby inhibiting the proliferation and migration of VSMCs and promoting their apoptosis (53). Another study indicated that lncRNA MEG8 as a ceRNA targeting the miR-195-5p/RECK (reversion inducing cysteine-rich protein with kazal motifs) axis attenuated the hypoxia-induced overproliferation, inflammation, and migration of VSMCs (54). However, overexpression of metastasis associated with lung adenocarcinoma transcript 1 (MALAT1) can also sponge miRNA-124-3p to positively regulate PPARα levels, inhibit proliferation, and promote apoptosis of VSMCs (55). Another study showed that the expression of p53, lincRNA-p21, and sirtuin 7(SIRT7)was downregulated, whereas that of miR-17-5p was upregulated in carotid tissue from AS mice and peripheral blood from patients with AS. p53-dependent lincRNA-p21 could increase SIRT7 expression by binding to miR-17-5p, thereby inhibiting VSMC proliferation and promoting apoptosis, while reducing AS-vulnerable plaques and lipid accumulation in mice (22). Similarly, overexpression of lncRNA small nucleolar RNA host gene 7-003 (SNHG7-003) inhibited the proliferation, migration, and invasion of VSMCs by suppressing miR-1306-5p, which directly binds SIRT7, upregulates its expression, and downregulates the contractile marker α-SMA in VSMCs (56). In contrast, overexpression of lncRNA CAMK2D associated transcript 1 (C2dat1) promoted the proliferation and migration of VSMCs by repressing miR-34a, another member of the SIRT family, by detecting the expression of PCNA, and by wound healing to detect migration (57). LncRNA small nucleolar RNA host gene 12 (SNHG12) was significantly upregulated in ox-LDL-treated hVSMCs. Moreover, SNHG12 acts as a sponge for miR-7665p. Eukaryotic translation initiation factor 5A(EIF5A)is a direct target gene of miR-766-5, and EIF5A promotes the proliferation and migration of ox-LDL-induced hVSMCs. However, silencing lncRNA SNHG12 counteracts the effect of EIF5A. This demonstrates that silencing lncRNA SNHG12 blocks the proliferation and migration of hVSMCs by targeting the miR-766-5p/EIF5A axis (58). In another study, knockdown of lncRNA SHNG12 targeting miR-199a-5p/HIF-1α was shown to be involved in the pathophysiological process of AS by regulating the phenotype of VSMCs (59). LncRNA lymph enhancer-binding factor 1-antisense RNA 1 (LEF1-AS1) regulates the PTEN/PI3K/AKT signaling pathway in VSMCs by targeting miR-544 (60). LINC01123 is highly expressed in patients with carotid atherosclerosis and promotes cell proliferation and migration by regulating the miR-1277-5p/KLF5 axis in ox-LDL-induced VSMCs (61). Similarly, investigators found that in ox-LDL-induced VSMCs, LINC00341 expression was increased, whereas miR-214 expression was significantly decreased. LINC00341 promoted FOXO4 protein expression by adsorbing miR-214, and forkhead box O4 (FOXO4) protein could counteract the promoter region of LINC00341 binding to promote its transcription, and LINC00341 promoted the proliferation and migration of VSMCs by regulating the miR-214/FOXO4 axis (62).

### LncRNA and diabetes

It is well known that diabetes can cause extensive damage to the macrovascular and microvascular systems in different organs and tissues, resulting in macrovascular complications like atherosclerosis, hypertension and stroke, and microvascular complications like diabetic nephropathy, diabetic retinopathy, and diabetic neuropathy (90). Therefore, it is important to understand that diabetes-related LncRNAs affect the development of diabetes by regulating smooth muscle cell phenotypic transition.

VSMCs are the main cells involved in the process of medial membrane vascular calcification. Calcified vascular smooth muscle cells can change from a contractile phenotype to a bone/chondrogenic phenotype (91). High glucose induces severe calcification/senescence in HA-VSMCs, a process that is exacerbated by lncRNA-ES3 (LINC00458) expression. Investigators found that lncRNA-ES3 acts as a ceRNA for miR-95-5p, miR-6776-5p, miR-3620-5p, and miR-4747-5p, exacerbating calcification/senescence in HA-VSMCs. Basic helix-loop-helix family member e40 (Bhlhe40) attenuates high glucose-induced calcification/senescence in HA-VSMCs by binding to the promoter region of the lncRNA-ES3 and subsequently regulating its expression in HA-VSMCs (63). Another study showed that lncRNA erythroid pro-survival (lincRNA EPS) could regulate the Wnt/β-catenin pathway by promoting TGF-β expression and interfering with Wnt3 and β-catenin expression, thereby inhibiting the differentiation to osteogenesis and migration of VSMCs and thereby reducing diabetes-related vascular calcification (64).

A study showed that lncRNA urothelial carcinoma associated 1 (UCA1) was significantly downregulated and miR-582-5p was upregulated in VSMCs and serum exosomes of patients with T2DM. There was a negative correlation between them and miR582-5p was a direct target of lncRNA UCA1. Downregulation of lncRNA UCA1 attenuated the proliferation and invasion of VSMCs induced by increasing glucose dose. However, these inhibited trends were partially abolished by co-transfection of miR582-5p inhibitor; therefore, the authors concluded that miR-582-5p was engaged in the repair of VSMCs induced by lncRNA UCA1 in the hyperglycemic state (65).

### LncRNA and hypertension

AngII is an active downstream peptide of the renin–angiotensin system that promotes the proliferation and migration of VSMCs by binding to its receptor (92). VSMCs are the major cellular components of the arterial mesothelium, and their proliferation promotes vascular remodeling in hypertension (93). Vascular remodeling caused by essential hypertension is a major cause of death in patients. Therefore, inhibiting cellular dysfunction and phenotypic transition in VSMCs may be a novel therapeutic strategy for essential hypertension.

The expression of serum lncRNA HLA complex group 18 (HCG18) was reduced in hypertensive patients and PDGF-BB-treated VSMCs. After knockdown of HCG18, the expression levels of contractile phenotypic markers, α-SMA, SM22α, and smoothelin, were significantly reduced in VSMCs, whereas synthetic markers, such as OPN, were increased; that is, knockdown of lncRNA HCG18 promoted the proliferation of VSMCs (67). In PDGF-BB-treated VSMCs, lncRNA growth arrest-specific transcript 5 (GAS5) blocked the PDGF-BB-induced proliferation and migration of VSMCs by competitively binding to miR-21, thereby attenuating its inhibitory effect on programmed cell death 4 (PDCD4). Thus, the lncRNA GAS5/miR-21/PDCD4 axis may be a potential target for hypertension treatment (68).

AngII-treated VSMCs were very close to the *in vitro* hypertensive state, and the apoptosis rate of VSMCs increased significantly after H_2_O_2_ treatment. Knockdown of lncRNA MRAK048635_P1 reversed this change. α-SMA, SM22α, and calponin expression levels were significantly reduced, while OPN expression levels were enhanced. VSMC proliferation and migration also increased. Knockdown of lncRNA MRAK048635_P1 in VSMCs also resulted in the overexpression of cyclin D1, cyclin E, CDK2, and CDK4. In the G1 phase, CDK phosphorylates the Rb protein and activates transcription factor E2F, which regulates the cell cycle and thus promotes the transcription of proliferation-related genes. Therefore, downregulation of lncRNA MRAK048635_P1 expression induces a phenotypic shift from contractile to synthetic VSMCs, promotes VSMC proliferation and migration, and inhibits apoptosis (70).

lncRNA AK098656 is mainly expressed in HASMCs, and lncRNA AK098656 overexpression promotes the proliferation of HASMCs by downregulating α-SMA and upregulating the expression of OPN and collagen-I. LncRNA AK098656 binds directly to both MYH11/FN1 (fibronectin-1) proteins, which can act as a scaffold to drag MYH11 closer to the proteasome to promote its degradation, and it can also mediate MYH11/FN1 degradation through the lysosomal pathway. Expression of MYH11, FN1, and α-SMA was also lower in the thoracic aorta, left renal artery, and superior mesenteric artery of rats overexpressing the lncRNA AK098656 gene, while collagen-I deposition increased, arterial lumen narrowing increased intima-media thickness and intima-media/lumen ratio and reduced vasodilation, which induced resistance to vascular arterial remodeling. LncRNA AK098656 can promote hypertension by accelerating contractile protein degradation, increasing VSMC synthetic markers, and, ultimately, antiatherogenic narrowing (71).

### LncRNA and revascularization, vascular remodeling

In a rat balloon injury model of restenosis, the expression of lncRNA H19 and miR-675 increased significantly in the neoplastic endothelium. The lncRNA H19-derived miR-675 was found to regulate PTEN and promote the proliferation of VSMCs by directly targeting the 3′-UTR of PTEN (42). In in-stent restenosis patient sera, lncRNA cyclin-dependent protein kinase inhibitors antisense RNA 1 (CDKN2B-AS1) levels were elevated, and miR-143-3p levels were decreased. In human carotid artery smooth muscle cells (hHCtASMCs), knockdown of lncRNA CDKN2B-AS1 resulted in the inhibition of cell proliferation and migration. miR-143-3p is a target of lncRNA CDKN2B-AS1. The results of *in vitro* studies suggest that the lncRNA CDKN2B-AS1/miR-143-3p axis may regulate the proliferation and migration of hHCtASMCs (72). Similarly, knockdown of lncRNA colorectal neoplasia differentially expressed (CRNDE] significantly inhibited PDGF-BB-induced proliferation and migration of VSMCs (73).

LncRNA GAS5 expression was reduced in PDGF-BB-induced VSMCs, but lncRNA GAS5 overexpression inhibited VSMC proliferation, blocked the cell cycle in the G1/G0 phase, and enhanced caspase-3 cleavage, promoting cell cycle arrest and apoptosis. Overexpression of lncRNA GAS5 increases the expression of the transcriptional regulator p53 and its downstream genes NOXA and p21. This hypothesis was supported by animal experiments. LncRNA GAS5 inhibited neoplastic endothelial formation by increasing the expression of p53 and its downstream genes NOXA and p21 to suppress VSMC proliferation and induce their apoptosis (69). LncRNA nuclear paraspeckle assembly transcript 1 (NEAT1) expression was elevated in PDGF-BB-induced VSMCs, and knockdown of lncRNA NEAT1 decreased the proliferation and migration ability of VSMCs and significantly reduced neoplastic endosomes, with similar changes in the proliferation markers Ki-67 and SM α-actin. PDGF-BB can also promote the binding of lncRNA NEAT1 to WDR5 to activate gene transcription and shift the SM-specific gene promoter from “open” to “closed” to suppress the expression of specific genes in SMCs, thereby regulating their phenotypic transition (75).

### LncRNA and pulmonary arterial hypertension

Pulmonary arterial hypertension (PAH) is a refractory cardiovascular disease characterized mainly by increased pulmonary vascular resistance and pulmonary artery pressure, resulting in vascular remodeling, leading to right ventricular hypertrophy, and eventually right heart failure. Hypoxia is a major factor in PAH pathogenesis. During hypoxic exposure, pulmonary artery smooth muscle cells (PASMCs) undergo excessive proliferation and migration, leading to hypertrophy of PASMCs and narrowing of the pulmonary vascular lumen, resulting in pulmonary hypertension (94).

In hypoxic PASMCs, lncRNA hoxa cluster antisense RNA 3 (Hoxaas3) affects transcriptional regulation by regulating histone H3K9 acetylation, which activates Hoxaas3 upregulation in PASMCs and increases the percentage of cells in the S + G2/M phase. In contrast, knockdown of Hoxaas3 reduces the number of cells in the S + G2/M phase and downregulates PCNA, Ki-67, cyclin A, D, and E expression, thereby inhibiting the proliferation of PASMCs under hypoxic conditions. Overexpression of Hoxa3 can reverse these changes. These results suggest that under hypoxia, Hoxaas3 regulates cell cycle changes by interacting with Homeobox a3 (Hoxa3) to allow PASMCs to proliferate (77). In hypoxia-induced PASMCs, the expression of another lncRNA, ANRIL, is significantly downregulated. The downregulation of lncRNA ANRIL expression caused more PASMCs to move from the G0/G1 phase into the G2/M+S phase, with increased expression of the cell cycle-related proteins, cyclin A, D, and E, and enhanced cell proliferation. In addition, the Transwell migration assay confirmed that the downregulation of ANRIL expression increased the migration of PASMCs under hypoxic conditions (25). A novel LncRNA TCONS_00034812 expression was significantly downregulated in PAH rats and hypoxic pulmonary artery SMCs. LncRNA TCONS_00034812 knockdown similarly increased the percentage of G2/M+S phase cells in PASMCs, ultimately leading to thickening of the pulmonary vascular mesoderm. Storkhead box 1 (STOX1) factor is a downstream lncRNA TCONS_00034812 target, and lncRNA TCONS_00034812 negatively regulates STOX1 to affect the proliferation of PASMCs (78). LncRNA Rps4l expression was downregulated in both hypoxia-induced PH tissues and PASMCs, and hypoxia increased the proportion of cells in the G2/M+S phase. In contrast, overexpression of lncRNA Rps4l inhibited the proliferation of PASMCs and attenuated hypoxia-induced cell cycle progression, causing PASMCs to stagnate in the G0/G1 phase. The increased expression levels of cyclins A, D, and E under hypoxic conditions were reversed by overexpression of lncRNA Rps4l. Upregulation of lncRNA Rps4l expression results in a significant reduction in the migratory capacity of PASMCs under hypoxic conditions by regulating the interleukin enhancer-binding factor 3 (ILF3)/HIF-1α axis (79). The expression of LncRNA AC068039.4, which functions in the same way, is significantly upregulated in hypoxia-induced PASMCs, and knockdown of lncRNA AC068039.4 reduced hypoxia-induced G2/M and S-phase cell percentages and attenuated PASMCs proliferation and migration. LncRNA AC068039.4 also binds miR-26a-5p through the ceRNA pattern to regulate the downstream target gene transient receptor potential canonical 6 (TRPC6) to promote PASMCs proliferation, migration, and cell cycle progression, thereby promoting pulmonary vascular remodeling (80).

The smad pathway is important for the vascular development and differentiation of VSMCs, which requires the phosphorylation of smad transcription factors for its subsequent nuclear translocation, DNA binding, and eventual transcriptional activation. Investigators found that the downregulation of lncRNA MYOSLID attenuates TGF-β1-induced Smad2 phosphorylation, disrupts F-actin formation, and blocks TGF-β1-induced megakaryoblastic leukemia 1 [MKL1) nuclear translocation, suggesting that lncRNA MYOSLID plays a key role in SMAD activation and subsequent transcription of VSMCs, and this study shows that lncRNA MYOSLID promotes the expression of contractile markers by inhibiting the proliferation and migration of VSMCs, but its effect on contractile gene expression in VSMCs is cellular context-dependent and may be restricted to VSMCs (81).

In hypoxia-treated PASMCs and PAH patient sera, investigators found a higher expression level of lncRNA NEAT1, which targets miR-34a-5p, while miR-34a-5p targets KLF4. Hypoxia significantly decreased αSMA and caspase-3 expression and increased PCNA and MMP-2 levels. In contrast, the knockdown of lncRNA NEAT1 reversed these alterations by the adsorption of miR-34a-5p and downregulation of KLF4, thereby slowing the progression of PAH (76).

LncRNA UCA1 was overexpressed in hypoxic HPASMCs, and overexpression of the inhibitor of growth proteins5 (ING5) reduced PCNA expression, inhibited cell viability, and promoted apoptosis in hypoxic HPASMCs, which was reversed by lncRNA UCA1 overexpression. LncRNA UCA1 competes with ING5 for heterogeneous nuclear ribonucleoprotein I, a protein that binds RNA and splice mRNA, and promotes proliferation and inhibit apoptosis (66). In PASMCs of hypoxia-induced rats, the expression of lncRNA CASC2 was significantly reduced and the expression of phenotypic transition markers troponin and α-SMA was reduced, while the amount of syndecan-1 and PCNA was significantly increased, and overexpression of lncRNA CASC2 resulted in opposite changes in the above markers. Therefore, overexpression of lncRNA CASC2 alleviated hypoxia-induced cell proliferation and migration, thereby regulating phenotype transition in PASMCs to partially restore hypoxia (51).

### LncRNA and aneurysm

Aortic aneurysms are usually defined as localized dilatations larger than 50% of the normal diameter and can occur in the thorax, but have the highest incidence in the abdominal aorta (95). Many inflammatory factors, such as CC chemokine ligand 2 (CCL2), IL-6, IL-1β, and TNFα, induce a chronic inflammatory response, inflammatory cell infiltration accompanied by elastin disruption and degeneration, and loss of mesangial SMCs. The pathophysiological process of aortic aneurysms is characterized by inflammatory cell infiltration, elastic and collagen fiber degradation, smooth muscle cell death, arterial wall defects, and increased oxidative stress (96). There is growing evidence that lncRNA promotes the proliferation of VSMCs or inhibiting apoptosis can prevent aneurysm progression.

In the thoracic aortic tissue of patients with aortic dissection (AD), lncRNA H19 was highly expressed, which competitively bound and inhibited the expression of miR-193b-3p. Upon PDGF-BB induction, the expression of lncRNA H19, MMP-2, and MMP-9 was upregulated; the expression of miR-193b-3p, α-SMA, and SM22α was downregulated; and the proliferation and migration rates of HASMCs were increased. However, silencing lncRNA H19 reversed the change induced by PDGF-BB. These results were consistently validated in animal experiments, indicating that silencing lncRNA H19 significantly attenuated PDGF-BB-induced proliferation and migration of HASMCs through the upregulation of miR-193b-3p, thereby reducing pathological injury in the thoracic aorta of AD mice (43). LncRNA X-inactive-specific transcript (XIST] is upregulated in the aortic wall tissue of patients with Stanford type A aortic dissection (TAAD) and correlates with the prognosis of TAAD. Knockdown of lncRNA XIST regulates downstream PTEN by inhibiting miR-17, which increases PCNA expression, accelerates Bcl-2 expression, and suppresses the levels of Bax and caspase-3, thereby promoting VSMC proliferation and inhibiting apoptosis to slow TAAD progression (49). Tissues near endothelial tears in patients with AD were proliferating; the expression of linc01278 and ACTG2 was downregulated; miR-500b-5p expression was upregulated; VSMC differentiation markers SMA, SM22α, calponin, and MYH11 were decreased. Silencing linc01278 targeted miR-500b-5p and ACTG2 in the three untranslated regions decreased the expression of SMA, SM22α, calponin, and MYH1; promoted the phenotypic conversion of aortic VSMCs from contractile to synthetic phenotypes; and promoted VSMC proliferation and migration. Thus, the linc01278/miR-500b-5p/ACTG2 axis may provide novel molecular mechanisms for diagnostic markers and therapeutic targets of AD (82). In AD, another lncRNA, PVT1 expression was upregulated, while the downregulation of lncRNA plasmacytoma variant translocation 1 (PVT1) expression led to an increase in α-SMA and SM22α expression and decreased MMP-2 and MMP-9 expression by targeting miR-27b-3p, which inhibited phenotypic transition and suppressed proliferation and migration in PDGF-BB-treated HASMCs (83).

In SMCs, the lncRNA lung cancer-associated transcript 1 (LUCAT1) exhibits anti-proliferative and pro-apoptotic effects, and knockdown of LUCAT1 leads to decreased caspase-3 activity and recovery after myelin regulatory factor (MYRF) overexpression. LUCAT1 acts as a decoy for miR-199a-5p and promotes MYRF expression, and lncRNA LUCAT1/miR-199a-5p/MYRF regulates the proliferation and apoptosis of SMCs in abdominal aortic aneurysms (85). In abdominal aortic aneurysm (AAA) tissues, lncRNA PVT1, and NCK-associated protein 1-like (NCKAP1L) expression was elevated and induced *in vitro* in AAA models, while miR-3127-5p showed the opposite trend, and lncRNA PVT1 acted as a sponge for miR-3127-5p to regulate NCKAP1L expression, inhibit VSMC proliferation, and induce apoptosis (84). In contrast, in AAA tissue, lncRNA SNHG5 was downregulated; overexpression of lncRNA SNHG5 could act as a molecular sponge for miR-205-5p and downregulate its expression, but upregulate the expression of SMAD4, thus increasing proliferation and migration and decreasing apoptosis in abdominal aortic aneurysm VSMCs (86). Another study found that H_2_O_2_ inhibited the activity of VSMCs, thus mimicking the AAA model. After H_2_O_2_ treatment, LINC00473 expression was upregulated, Bax expression was enhanced, and Bcl-2 expression was decreased. In AAA, brain acid-soluble protein 1 (BASP1) expression was inversely correlated with miR-212-5p expression but positively correlated with LINC00473 levels. These results suggest that LINC00473 competitively interacts with miR-212-5p to promote BASP1 expression and VSMC apoptosis, ultimately leading to AAA exacerbation (87). In AAA tissues and AngII-stimulated VSMCs, the expression of lncRNA CRNDE was downregulated, and the data suggest that overexpression of lncRNA CRNDE can promote VSMC proliferation and inhibit apoptosis by upregulating Bcl-3 ubiquitination of Smad3 protein and upregulating smad3 expression, thereby inhibiting mouse AAA growth (74).

## Conclusion and perspectives

LncRNAs are relatively newly discovered RNA molecules with important regulatory functions. These findings suggest that lncRNAs may have profound effects on the regulation of VSMCs and are regulators of gene expression and vascular function. Although our knowledge of lncRNAs is limited, their emergence may further our understanding of the complex regulatory network of cellular function in clinical vascular diseases. Targeting lncRNAs may be an extremely promising modality of governance not only in tumors but also in cardiac or vascular diseases, and thus, they are regulators of smooth muscle cell phenotypic transition.

## Author contributions

B-HL and H-BL: original draft writing and manuscript revision. D-XL and Z-GC: manuscript revision. S-XG and JZ: graphic design. G-AZ and FL: manuscript design and revision.

## Funding

This work was supported by the Research Projects of Higher Education Institutions in Henan Province of China (Nos: 21A320012 and 22A360017), Key Specialized Research and Development Breakthrough in Henan Province of China (Nos: 212102310350, 222102310442, and 222102310631), Graduate Student Research Innovation Support Program of Xinxiang Medical University in Henan Province of China (YJSCX202174Y), and The First Affiliated Hospital of Xinxiang Medical University Youth Foundation (Grant Nos. QN-2017-B026 and QN-2021-B15).

## Conflict of interest

The authors declare that the research was conducted in the absence of any commercial or financial relationships that could be construed as a potential conflict of interest.

## Publisher's note

All claims expressed in this article are solely those of the authors and do not necessarily represent those of their affiliated organizations, or those of the publisher, the editors and the reviewers. Any product that may be evaluated in this article, or claim that may be made by its manufacturer, is not guaranteed or endorsed by the publisher.
